# Comment on: “*Blockade of intercostobrachial nerve by an erector spinae plane block at T2 level*”

**DOI:** 10.1186/s40981-023-00653-5

**Published:** 2023-10-04

**Authors:** Raghuraman M. Sethuraman

**Affiliations:** Department of Anesthesiology, Sree Balaji Medical College & Hospital, BIHER, #7, Works Road, New Colony, Chromepet, Chennai, 600044 India

To the Editor,

I read with great interest the recently published article describing a case of brachial vein transposition-arteriovenous fistula establishment under an infraclavicular brachial plexus block and an additional erector spinae plane block (ESPB) at the T2 level to block the intercostobrachial nerve (ICBN). I wish to present my reflections on that case report [1].

While explaining the background for choosing this combination of blocks, Yoshida et al. [1] state that both the supraclavicular and infraclavicular approaches of brachial plexus blocks could block all the nerves of the upper arm except for the ICBN and cited two references [2, 3] to support that point. While this statement is correct for the supraclavicular approach, the infraclavicular approach mostly blocks the ICBN as this technique is performed in close proximity to the axilla [4], providing sensory coverage in about 80% of patients [5]. Furthermore, the two referenced studies [2, 3] by Yoshida et al. [1] do not defend that statement. Race et al. [2] observed that the medial cutaneous nerves of the arm can have a variable number of cutaneous branches, while Johnson et al. [3] only described the anatomical variations of the brachial plexus. Indeed, Johnson et al. stated that the infraclavicular part of the brachial plexus is located in the axilla topographically [3] which corroborates the most probable coverage of ICBN by an infraclavicular block [4, 5].

Hence, the combination chosen by Yoshida et al. [1] needs a careful analysis. Yoshida et al. [1] could have tested the sensory coverage of the ICBN after performing the infraclavicular block. Notably, Moustafa et al. excluded the patients in whom the preliminary infraclavicular block covered ICBN, although their statement that “any brachial plexus block definitely spares the ICBN” was contradictory to this method and misleading [6].

Because of the highly variable extra-thoracic anatomy of the ICBN [4], there is a possibility of an infraclavicular block sparing it, requiring an additional block. I suggest the method of blocking the ICBN in the axilla with 1 ml of local anesthetic as described by Thallaj et al. [7]. While discussing this point, Yoshida et al. [1] state that an accidental puncture of the vessel in the axilla might compromise the blood flow to the arteriovenous fistula. However, this complication is very rare under ultrasound guidance. Also, if we are worried about this rare possibility, then what about performing the infraclavicular block which also has major vessels surrounded by the cords of the brachial plexus? Indeed, more worrisome?

In conclusion, an infraclavicular block would suffice for this procedure mostly; ICBN block performed at the axilla can be added if required. ESPB has disadvantages such as excess local anesthetic, extra time, position-related complications, and cumbersome to the patient and operating room personnel, especially for this type of geriatric patient with severe co-morbid conditions.

## Data Availability

Not applicable.
